# The Relation of the Teeth to the Lips and Face

**Published:** 1898-10

**Authors:** A. O. Hunt

**Affiliations:** Chicago, Ill.


ARTICLE V.

### THE RELATION OF THE TEETH TO THE LIPS AND FACE.

DR. A. O. HUNT, CHICAGO, ILL.

In Illinois Society.

By close observation and careful records phrenology and physiognomy have been brought to such a stage of perfection that, by the outlines of the body, combined with its movements, it is possible to tell the character and disposition of an individual, as well as what those characteristics fit them for in the way of occupation. Palmistry also, by the same careful observation and correct records, can make some very close guesses as to those things which govern an individual, both past and present, by the lines, the hills and mountains as seen in the palm of the hand, to the shape and outlines of the fingers and thumbs.

Art and sculpture have by the same means been enabled to fix a classical standard of the ideal shape of the human form as seen in the external outlines on the surface of the body, exhibited by the position and action of the muscles. There has also been established a definite relation as to size in the circumference and length of parts of the body, as compared to each other.

There are also distinctions in the distance between the eyes, the length, width and position of the eyes, length of the nose, size of the mouth, etc. And yet these features are so affected by their surrounding conditions, that the human family is divided into five distinct races of people, and again for reasons into tribal and national characteristics, still retaining all the essentials of the human form. Wherever the study of the human form has been specialized, both anatomy and physiology (form and function) have been well considered. It has engaged the best minds, and produced an extensive and valuable literature, such as Darwin's "Expression of Men and Animals," Rimmer's "Art Anatomy," various works on phrenology and palmistry, etc.

The subject here presented is one I have been investigating for several years, and repeatedly demonstrated the conclusions arrived at in the field of prosthetic dentistry.

This investigation was based on the belief that if the parts of the body had so many fixed relations to each other that other specialists had found a basis on which to build, that the teeth must also have a definite relative position to other parts of the head and face. If this could be shown it would relieve the dentist of many discouragements, and assist the beginner in mastering the most difficult operation with which he has to deal. Instead of being obliged to wait for eight or ten years before he begins to feel interest in the work, or confidence in himself in the field of dental prosthesis, a set of principles might be established as a basis, so correct that he could proceed intelligently in the construction of an artificial denture without fear as to the final results.

The methods followed by which the conclusions were reached, as presented for your consideration, was to make sets of plaster models of all classes of natural dentures, making at the same time, and of the same individual, two plaster casts of the lower half of the face; one with the features at rest and one where the teeth were closely occluded, both being essential in the study of the subject. The casts of faces made in these two positions show some widely varying conditions.

Our interest is centered more particularly in the lower fourth of the face, or the maxillary region where most, if not all, of the muscles of expression concentrate.

The lips will demand our first consideration. I do not think it necessary to take up in detail each muscle and its function. Into the orbicularis oris muscle anastomose all the elevating and depressing muscles; in the upper lip all but one are elevating muscles; in the lower lip all but one are depressing muscles. In the one, the depressor ale nasi of the upper lip, it is not so much a muscle of depression as one of eversion and inversion; that is, it will move the lip inward or outward, but not downward to any extent, while the elevating muscle of the lower lip has a very great function. In other words, it is with difficulty that the upper lip can be forced down over the lower lip, and with some it cannot be done at all, while nearly every one can raise the lower lip over the upper, sometimes to the extent of touching the base the nose.

The lips assume many forms. The full lip, the thin lip, the flat lip, the pointed lip, the full upper lip and thin lower, and *vice versa*. In fact, the great law of variation is as constant here as elsewhere, and yet the change is never so great as to destroy the identity of the human characteristics.

The various changes of appearance are more apparent than real, as this appearance is controlled mostly, if not wholly, by the position of the teeth and shape of the alveolar process behind them. In following this investigation, if one expects to come on anything that may be regarded as typical either in the arrangement, size; form or occlusion of the teeth, they are doomed to disappointment; outside of the fact that they are human teeth and cannot be mistaken, there is nothing else typical.

Let us first consider the cuspids, the most important. They have a position distinctively their own, and but little variation is observed in that respect. In their development the position of the follicles are above the line of the follicles of the incisors or bicuspids. Their position is directly underneath the ale of the nose, between the fibers of the levator labii et aleque nasi and the levator labii superiors. In the process of development and eruption they retain this position between the muscles, and when finally in position the cusp or point is in a line with, and directly underneath, the outer margin of the ale of the nose. The only instances where this is not true (and they are rare), are where, by the premature eruption of the lateral incisor and first bicuspid, the two latter have come into close contact at the interproximal space. If, however, this contact is not close and there is room enough for the wedge-shaped lingual side of the cuspid to gain an entrance, the cuspid assumes its regular position, crowding the incisors and bicuspids either within or without the arch, or sometimes causing them to rotate in their alveoli.

Another instance where the variation may occur is where they erupt in the palatal bones, or they may be locked within the arch by the occlusion of the apposing teeth.

The mesio-labial margin usually forms the turn of the arch. The labial surface is presented mostly to the buccal aspect. The central and lateral incisors, unless some of them are abnormal in size (which is not unusual) occupy the space between the cuspids. The variation in the relative width of the incisors is shown by the common irregularity as to alignment. It is very rare not to find them either slightly within or without the arch or some of them rotated in the alveolus.

The position of the bicuspids is that they are nearly hidden behind the cuspids, if the median line is taken as the center of vision. In every movement of the levator anguli oris and the risorins muscles, inward pressure is brought to bear on these teeth. This pressure is augmented by the action of the levator and depressor anguli oris muscles, as well as by the anterior margin of the buccinator, in the act of opening the mouth. As the molars develop and erupt with the growth of the maxillary, they are farther removed from the action of so many muscles which changes the conditions materially. They are at liberty to follow the law of individuality which dominates all forms, and thus arrange themselves according to the temperament, whether basal or mixed in type.

The levator menti inferioris, the depressors labii inferioris and depressors anguli oris in the interlacing of their fibers present analogous forces to the muscles of the upper lip and cheeks, which operate to keep the inferior cuspids in their normal position.

The lower teeth are not so likely to be malpositioned when the upper teeth are in their normal places, except at the time of the eruption of the third lower molars.

In considering the lower teeth, we find the central incisors are always the length of the lower lip when the face is at rest. This is not likely to be the fact in the relation of the upper lip and upper teeth. Any unusual strength of the intermaxillary bones or a roundness or fullness to the superior alveolar border will cause what we call a short upper lip. The lip is shortened because when it is lifted above the rounded or full border of the process there are no muscles that readily act to bring the lip down over it; so much of an effort is required to accomplish this that the individual rarely exercises it. The result is, that many persons do not close the upper lips when the face is at rest, while at the same time the teeth may be very nearly in contact and the occlusal margins of the upper central incisors are touching the inner edge of the lower lip.

The variation in the profile of faces requires that some standard be used to decide whether the lip and teeth are of the same length.

If an instrument be passed between the lips at the parting at right angles to the plane of the lower fourth of the face, it will touch the upper central incisors at a point corresponding to the occiusal edges of the lower central incisors. If the teeth are separated slowly without disturbing the instrument or lower lip, it will be found that the point of the instrument will indicate the length of the lower teeth as stated.

The relation of the teeth to the lips and face are of such a positive character that there could not be a good and beautiful face without a correspondingly good arrangement of the teeth. It is possible for a close observer to tell by looking at the outlines of a face without seeing the teeth as to whether any of the latter are missing or out of position, and which are so.

If in the construction of artificial dentures the principles as presented are noted and marked on the wax used in taking the "bite," and such other notes made as cannot be recorded on the wax, the element of guessing will be eliminated and the years of experiment to obtain a good result will be shortened materially.

We have then the following as a summary:

When the face is at rest the teeth are slightly apart.

The occlusal edges of the central incisors are touching the inner margin of the lower lip.

The cusps of the superior cuspids are in a line vertically underneath the outer ale of the nose.

The length of the lower incisors is the length of the lower lip.—*Dental Brief.*

